# Reactive Byproducts of Plant Redox Metabolism and Protein Functions

**DOI:** 10.32607/actanaturae.27477

**Published:** 2024

**Authors:** E. I. Sharova, S. S. Medvedev

**Affiliations:** St Petersburg University, St. Petersburg, 199034 Russian Federation

**Keywords:** post-translational modifications (PTMs) of proteins, proteoforms, carbonylation, nitrosylation, glutathionylation, sulfenylation

## Abstract

Living organisms exhibit an impressive ability to expand the basic information encoded in their genome, specifically regarding the structure and function of protein. Two basic strategies are employed to increase protein diversity and functionality: alternative mRNA splicing and post-translational protein modifications (PTMs). Enzymatic regulation is responsible for the majority of the chemical reactions occurring within living cells. However, plants redox metabolism perpetually generates reactive byproducts that spontaneously interact with and modify biomolecules, including proteins. Reactive carbonyls resulted from the oxidative metabolism of carbohydrates and lipids carbonylate proteins, leading to the latter inactivation and deposition in the form of glycation and lipoxidation end products. The protein nitrosylation caused by reactive nitrogen species plays a crucial role in plant morphogenesis and stress reactions. The redox state of protein thiol groups modified by reactive oxygen species is regulated through the interplay of thioredoxins and glutaredoxins, thereby influencing processes such as protein folding, enzyme activity, and calcium and hormone signaling. This review provides a summary of the PTMs caused by chemically active metabolites and explores their functional consequences in plant proteins.

## INTRODUCTION

Living systems demonstrate a remarkable ability to substantially increase the basic information encoded within their genome as regards potential protein functionalities. The principal mechanisms involved here include, but are not limited to, alternative mRNA splicing [1, 2, 3] and post-translational modifications (PTMs) of proteins [4, 5, 6, 7]. PTMs of proteins, which encompass enzymatic or spontaneous alterations to amino acid residues, can dramatically modulate protein functions or lead to their loss. PTMs significantly increase the diversity and functionality of proteins, serving as a foundation for numerous cellular signaling processes.

Recent studies [4, 8, 9, 10, 11] have demonstrated an increasing preference for the term “proteoforms” to encompass the diverse modifications of a protein derived from a single gene. The term denotes protein isoforms originating from a single gene, exhibiting differences in splicing and PTMs [8, 9, 11]. Proteoforms encompass various mechanisms of biological variability (modification) a protein molecule undergoes, determining its functional specificity. Proteoform-level protein characterization is essential for a comprehensive understanding of the biological processes controlled by protein molecules. Protein functions are considerably altered by various PTMs, such as phosphorylation, N- and O-linked glycosylation, methylation, acylation, S-glutathionylation, ubiquitination, and sumoylation [7, 8, 11]. Furthermore, each protein usually posesses several PTM sites. As a result, the number of proteoforms can exceed the number of genes encoding these proteins by several orders of magnitude [8, 12]. Consequently, var ied PTM patterns within the same protein substantially increase proteoform heterogeneity [4, 8, 9, 11]. The production of diverse proteoforms from a single gene sequence constitutes an efficient strategy to expand the functional repertoire of the proteins that mediate plants response to changing environmental conditions [11]. A complete understanding of cellular physiological and biochemical processes at the protein level requires knowledge of the identity and functional specificity of these proteoforms.

Most chemical reactions occurring in the body are enzymatically controlled. However, it is possible for many metabolites to spontaneously react with each other and with the biomolecules that are crucial for homeostasis. Highly chemically reactive metabolites are of paramount importance, as they inflict rapid and frequently irreversible damage upon nucleic acids, lipids, carbohydrates, and proteins. Their impact on proteins is defined by the highest degree of complexity and variety [13]. For a long time, spontaneous reactions were thought to impede the well-regulated metabolism. It is now widely accepted that these reactions are fundamentally integrated within the mechanisms governing homeostasis under variable environmental pressures. Numerous PTMs serve as compelling examples illustrating the correlation between spontaneous and enzymatic processes [13, 14]. The strong electrophilic and oxidizing properties of reactive oxygen, nitrogen, and sulfur species and carbonyl-containing compounds are evident in their electron abstraction from carbon, sulfur, and nitrogen atoms and their addition to the nucleophilic groups within proteins [15]. Furthermore, a given active agent, for example the hydroxyl radical, may function as both an oxidant and an electrophile.

This field of study is characterized by rapid advancement necessitating frequent generalization. Our grasp of many phenomena remains incomplete, leading to conjectural interpretations. This review focuses on key findings that illuminate the modern concept of proteoforms produced by the reactive byproducts of plant redox metabolism.

## REACTIVE CARBONYL COMPOUNDS

Carbonyl compounds are organic molecules containing a carbonyl group (oxo group), C=O. While typically limited to aldehydes and ketones, carbonyl groups are also present in esters, amides, and other carboxylic acid derivatives. First and foremost, they are intermediates of the glycolysis, the pentose phosphate pathway, and the Calvin cycle [16, 17]. At high concentrations, these compounds can cause spontaneous protein glycation and damage, which they do in humans with diabetes [17]. At the same time, there are carbonyl compounds in cells that exhibit such activity even in micromolar concentrations.

Approximately 20 carbonyl compounds have been identified in plants. The most prevalent among these are the dialdehydes: glyoxal, methylglyoxal (MG), malondialdehyde (MDA), and α,β-unsaturated aldehydes, with 4-hydroxy-2-nonenal (HNE) being the most frequently encountered [16, 17]. The carbonyl groups of these compounds exhibit a high degree of polarization (C+=O-), facilitating the electrophilic attack on nucleophilic protein residues. Reactive oxygen species (ROS) induce lipid peroxidation, ultimately yielding glyoxal, MDA, and HNE as end products [18]. MG is the result of the spontaneous dephosphorylation of triose phosphates, namely dihydroxyacetone phosphate and glyceraldehyde-3-phosphate [19]. Plant cells usually exhibit MG concentrations under 10 μM [20], but stressful conditions, including phosphate starvation [20] and heavy metal contamination [21], induce substantial elevations in the MG content.

The toxicity of active carbonyl compounds to proteins is a result of their ability to attach to the amino groups of lysine and arginine, and the thiol group of cysteine. The outcome of this addition is the carbonylation of proteins, manifested as an augmented presence of carbonyl groups in their structure. When carbonylation results from the binding of sugars and their derivatives to proteins, the process is termed protein glycation [20, 22], a non-enzymatic PTM resulting from the interaction of proteins with sugars and the carbonyl products of their degradation [17].

The mechanism of glycation, first studied over 100 years ago as a phenomenon of protein fructosylation during food preparation, is now known as the Maillard reaction. At elevated temperatures, spontaneous glucose and fructose degradation products bind to the ε-amino groups of protein lysine residues, forming Schiff bases that subsequently undergo Amadori rearrangement [23].

**Fig. 1:**
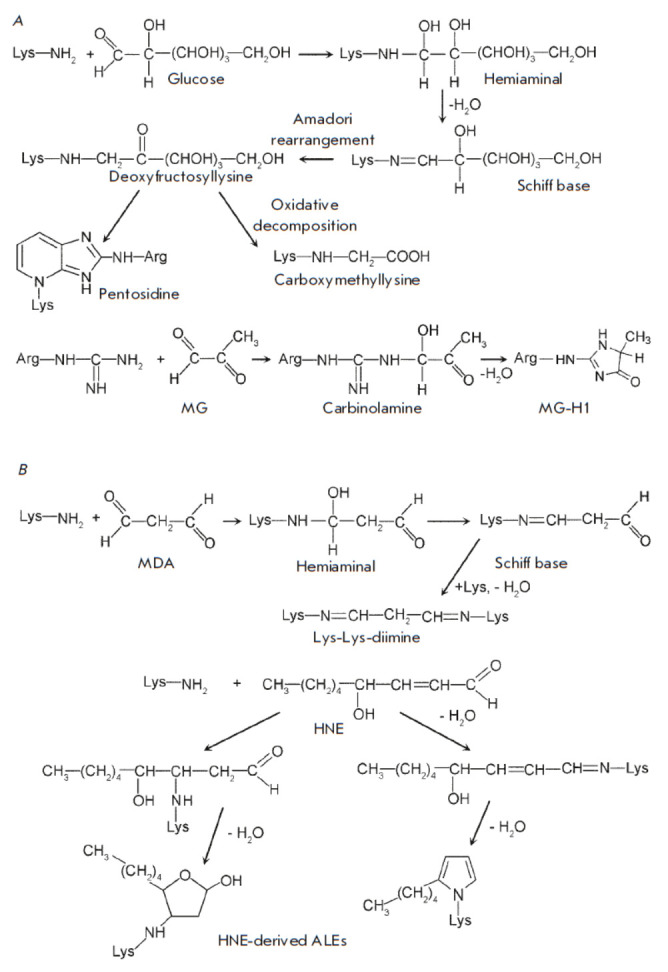
Protein carbonylation. (*A*) Glycation by glucose and methylglyoxal (MG), (*B*) Lipoxidation by malondialdehyde (MDA) and 4-hydroxy-2-nonenal (HNE)

Similar processes are observed within living cells. Glucose and its oxidation products can launch an electrophilic attack on the ε-amino group of lysine (*Fig. 1A*). As a result, an unstable primary glycation product, a hemiaminal, is formed, with the glycation process being reversible at this stage. However, dehydration of the hemiaminal leads to a Schiff base formation, which then rapidly undergoes Amadori rearrangement, resulting in deoxyfructosyllysine. Further spontaneous reactions lead to the intracellular accumulation of advanced glycation end products (AGEs). AGEs classification is commonly predicated on their carbonyl precursors and/or intermediates [24]. AGEs exhibit significant structural heterogeneity, encompassing diverse aliphatic, aromatic, and heterocyclic moieties [17]. Carboxymethyllysine constitutes the most prevalent product of the Maillard reaction. The pentosidine cross-linking between modified lysine and arginine residues also serves as an indicator of protein glycation [25].

Glyoxal and MG exhibit activity a thousand times higher than that of glucose [20]. Their main target is the guanidine group of arginine, with which they form a carbinolamine (*Fig. 1A*) that is spontaneously converted into a series of hydroimidazolone derivatives: G-H (glyoxal-derived hydroimidazolone) and MG-H (methylglyoxal-derived hydroimidazolone) [26, 27]. In plants, MG-H1 is the most abundant AGE [20].

When the products of free-radical oxidation of lipids serve as carbonylation agents, then protein lipoxidation occurs [28]. While this modification is not inherently oxidative, it frequently exacerbates the damage to the protein under oxidative stress conditions. The accumulation of advanced lipoxidation end products (ALEs) results from the spontaneous transformations of unstable primary adducts, which exhibit a range of characteristic chemical structures within proteins [29]. The proteins involved in basic metabolic pathways, signal transduction, cytoskeletal structure, and transcriptional control are all targets of lipoxidation.

The end products of the free-radical oxidation of lipids actively attack lysine residues [27]. The interaction between MDA and lysine results in the formation of a hemiaminal, which is promptly converted to a Schiff base (*Fig. 1B*). The interaction of the second aldehyde group of MDA with a lysine residue of the same or another protein results in cross-linking in the form of lysine-lysine diimine, a common ALE [30]. The attachment of HNE and other α,β-unsaturated aldehydes to lysine residues in proteins occurs via the Michael addition (*Fig. 1B*) [27, 29]. Among the most significant hallmarks of protein damage resulting from lipid peroxidation are HNE-derived heterocyclic protein adducts.

Plant protein glycation and lipoxidation significantly augment under stressful conditions [17, 20, 31]. Given the irreversible nature of these alterations, the principal survival strategy of organisms involves antioxidant- mediated prevention of lipid peroxidation and enzymatic detoxification of MG and glyoxal by glyoxalases.

**Fig. 2:**
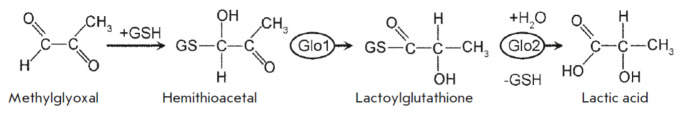
Detoxification of methylglyoxal (MG) by Glo1 and Glo2 glyoxalases

Glyoxalases convert MG into lactic acid (*Fig. 2*) and glyoxal into glycolic acid [24]. The reactions proceed with glutathione (GSH) functioning as a cofactor. The spontaneous reaction between MG and the sulfhydryl group of GSH produces a hemithioacetal. Glyoxalase I (Glo1) catalyzes the isomerization of this adduct to lactoylglutathione, which is then hydrolyzed by glyoxalase II (Glo2). The presence of glyoxalases has been documented across a wide range of prokaryotic and eukaryotic organisms. In Arabidopsis, 22 genes encoding Glo1 and 9 genes encoding Glo2 have been identified. These enzymes are the most active within chloroplasts. However, their presence has also been observed in mitochondria, nuclei, cytosol, cell walls, and peroxisomes [32].

Irreversible protein carbonylation occurs throughout the plant life cycle and is widely considered an unavoidable process of protein damage, aggravated by stress. It is evident that our understanding of the functional aspects of protein carbonylation lags considerably behind the progress made in its chemical study. Published data suggest that protein carbonylation is subjected to fine regulation and is involved in hormonal signaling, seed germination, flowering, and other processes, rather than being solely dependent on the reactive carbonyl compounds level [33].

## REACTIVE NITROGEN SPECIES

Reactive nitrogen species are formed as a result of spontaneous redox transformations of nitric oxide •NO and a number of other nitrogen-containing substances. The involvement of reactive nitrogen species in plant growth, stress response, and hormone signaling has gained significant attention in recent years [34, 35, 36, 37].

The biosynthesis of •NO in mammals involves the conversion of arginine by nitric oxide synthases. These enzymes are NADPH-dependent oxygenases with flavin, iron-porphyrin, and tetrahydrobiopterin as essential cofactors. The function of NO synthases extends beyond •NO synthesis to include the targeted nitrosylation of proteins, achieved through protein- protein interactions [38].

**Fig. 3:**
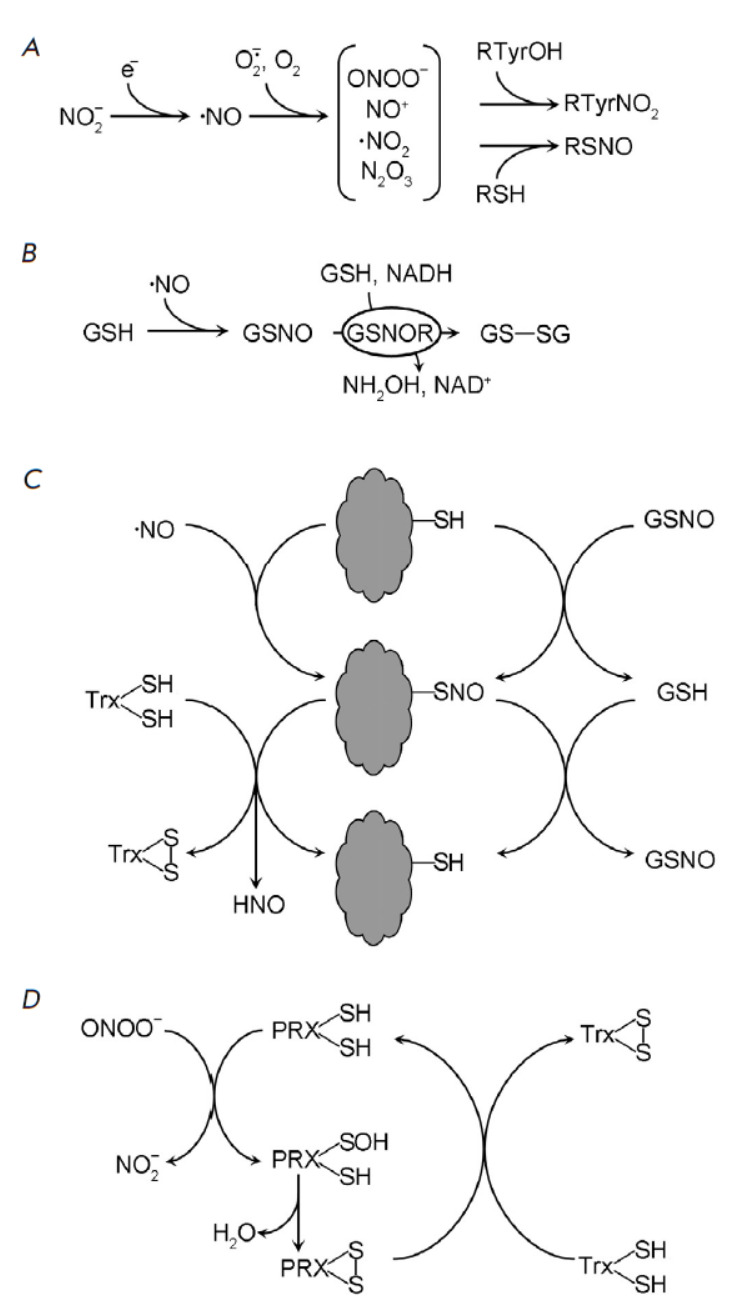
The effect of reactive nitrogen species on proteins. (*A*) The general scheme of nitric oxide (•NO) formation, its conversion into chemically active species and incorporation into proteins, (*B*) Glutathione (GSH) nitrosylation and nitrosoglutathione (GSNO) denitrosylation with nitrosoglutathione reductase (GSNOR), (*C*) Nitrosylation, transnitrosylation and denitrosylation of proteins, (*D*) Utilization of peroxynitrite (ONOO–) with peroxyredoxins (PRX). Trx – thioredoxins

Peroxynitrite ONOO-, nitrosonium cation NO+, nitrogen dioxide •NO_2_, etc., interact readily with proteins (*Fig. 3A*). NO-dependent protein PTMs of biological significance involve the nitrosylation of transition metals, S-nitrosylation of cysteine residues, and tyrosine nitration [40]. S-nitrosylation serves a crucial regulatory function. Therefore, disruption of its activity in the human body is associated with severe neurodegenerative diseases, immune system impairment, and cardiovascular dysfunction [38]. In plants, S-nitrosylation affects enzymatic activity, subcellular localization, proteolytic degradation rates, and protein- protein/protein-DNA interactions [34, 41, 42].

Protein nitration is primarily inflicted by ONOO-, while S-nitrosylation is predominantly mediated by nitrosoglutathione (GSNO), which is generated through the reaction of GSH with reactive nitrogen species (N_2_O3, NO+) (*Fig. 3B*). GSNO serves as a storage and transport form of•NO within plant cells [43]. Spontaneous transnitrosylation reactions transfer •NO from GSNO to the thiol groups of proteins (*Fig. 3C*).

GSNO denitrosylation is a function of the activity of nitrosoglutathione reductases (GSNORs), which are conserved proteins found in the cytoplasm and nucleoplasm [44]. The denitrosylation of SH-groups of proteins (R-SNO → R-SH) is achieved through the action of thioredoxins (Trx) or via GSH transnitrosylation (*Fig. 3C*). Along with GSNOR and Trx, reactive nitrogen species detoxification is facilitated by peroxiredoxins (PRX), which catalyze the conversion of peroxynitrite to nitrite (*Fig. 3D*) [34, 45]. This process yields a reduced thiol protein (R-SH) and oxidized Trx, with the latter undergoing reduction by NADPH-dependent Trx reductase. Transnitrosylation is catalyzed by a transnitrosylase possessing an SNO moiety, which facilitates the transfer of the •NO to the target protein [34].

In both plants and animals, nitration typically leads to the proteins damage and subsequent degradation. Particularly susceptible to nitration are catalase and the enzymes of the ascorbate-glutathione cycle, the main participant in ROS removal in plants [46].

Due to its lipophilic nature and ability to readily cross membranes, the free radical •NO serves as an effective signaling molecule in autocrine and paracrine cellular communication. The signaling role of •NO has been extensively investigated in studies of mammals and humans [38]. Guanylate cyclase, a key •NO receptor, is known to undergo nitrosylation of its heme iron (Fe^2+^), forming FeNO. The enzyme activated through this modification produces cyclic GMP, which functions as a secondary messenger [47].

The sensitivity to nitric oxide is an evolutionarily conserved characteristic of hemoproteins with H-NOX (Heme-nitric oxide/oxygen binding) domains. Domains with the ability to serve as •NO sensors have been detected in bacteria, fungi, and animals, including humans [48, 49]. It used to be believed that these proteins were absent in plants; however, recent research has demonstrated the existence of several •NO-sensitive hemoproteins in plants. Hemoproteins in plant organisms sensitive to •NO were discovered as possessing conserved H-NOX domains that can bind both •NO and O_2_. Several signaling pathways utilizing these proteins as sensors for •NO or O_2_ have been characterized. Specifically, plant hemoproteins with H-NOX domains have been demonstrated to mediate crucial •NO-dependent processes, including pollen tube growth and stomatal closure [51].

The understanding of •NO-signaling pathways in plants is yet to be fully expanded. A dearth of reliable data exists regarding the functions of cyclic GMP and nitrosylation of the protein heme and non-heme iron. At the same time, the impact of S-nitrosylation on the enzymatic activity, subcellular localization, proteolysis rate, and protein-protein interactions affecting the proteins of the basic metabolism has been established [52]. The activating (+) and inhibitory (–) effects of S-nitrosylation were confirmed for enzymes that regulate the balance of ROS in plant cells: superoxide dismutase (–), catalase (–), ascorbate peroxidase (+), mono- and didehydroascorbate reductases (–).

The process of S-nitrosylation influences the proteins that participate in hormone signaling [47, 53]. In Arabidopsis seeds, the accumulation of •NO during imbibition leads to the S-nitrosylation and proteasomal degradation of ABI5, a transcription factor crucial for abscisic acid (ABA)-dependent gene expression [42]. Consequently, ABA signaling is suppressed, thereby stimulating seed germination. In ABAdependent stomatal closure, •NO appears to mediate the termination of this process by suppressing ABA signaling; this is achieved via nitration/S-nitrosylation of the PYR1 hormone receptor [54] and the SnRK2.6 protein kinase, both crucial components of ABA signaling [55].

The effect of •NO on gibberellin and auxin signaling in Arabidopsis has been reported. The conserved cysteine residue within the DELLA protein RGA has been demonstrated to undergo S-nitrosylation, thus inhibiting the proteasomal degradation of this negative regulator of gibberellin signaling [56]. S-nitrosylation-mediated prevention of Aux/IAA17 proteolysis leads to the suppression of auxin signaling [57].

So, the information on the effects of reactive nitrogen species on plant proteins largely describes the mechanisms and roles of S-nitrosylation. The existing literature on transition metal nitrosylation within proteins is scarce, notwithstanding the discovery of plant proteins containing NO-sensitive H-NOX domains [50].

## REACTIVE OXYGEN SPECIES

Redox transitions O_2_ ↔ H_2_O in living organisms invariably produce various ROS, including O_2_ •-, H_2_O_2_, •OH, 1O_2_, capable of direct interaction with proteins.

Molecular oxygen typically exists in a relatively unreactive triplet state (3O_2_). The formation of ROS occurs through enzymatic and non-enzymatic processes, specifically within the mitochondrial and chloroplast electron transport chains, peroxisomes during photorespiration, cell walls during hypersensitive responses, and in the cytoplasmic and nucleoplasmic compartments. 3O_2_ is activated via two primary mechanisms: 1) increase in the energy of one of the electrons and appearance of the active singlet form of oxygen 1O_2_ under the influence of photosensitizers (mainly excited triplet chlorophyll 3P680*) and UV radiation; and 2) reduction of one of the 3O_2_ atoms and its transformation into a superoxide anion radical (O_2_ •-) by metals with variable valency or organic electron donors [58]. In acidic environments (vacuoles, cell walls), O_2_ •- is protonated and converted to the hydroperoxyl radical (HO_2_ •). Hydrogen peroxide (H_2_O_2_) is a product of the activity of superoxide dismutases, plant class III peroxidases, amine oxidases, and oxalate oxidases, as well as spontaneous transformations of HO_2_ • and O_2_ •-. The formation of the hydroxyl radical •OH occurs by the Fenton reaction from H_2_O_2_ with the participation of transition metals: H_2_O_2_ + Fe^2+^(Cu^+^) → •OH + Fe^3+^(Cu^2+^) + OH-.

The high reactivity of ROS results in reactions with proteins, lipids, carbohydrates, and nucleic acids. Highly reactive oxygen species, such as (HO_2_ • and •OH), initiate chain reactions resulting in the generation of numerous free radicals, thereby inducing biomolecular degradation [59].

**Fig. 4:**
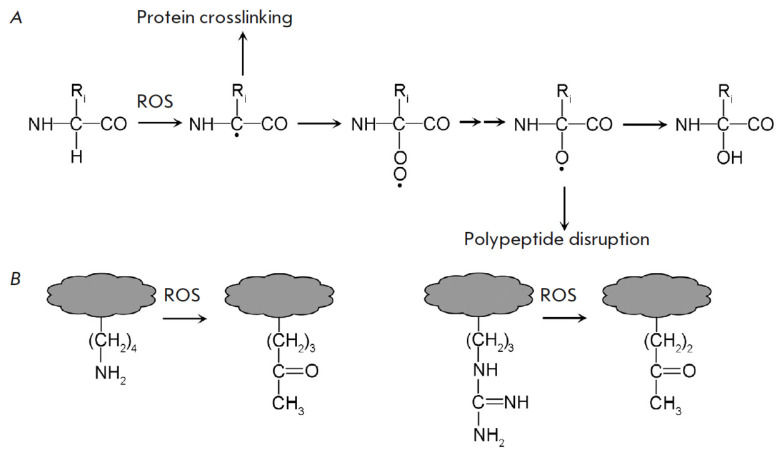
Irreversible oxidation of the polypeptide chain (*A*) and amino acid side chains (*B*) under the action of ROS

ROS selectivity is inversely correlated with their activity. Thus, both the main protein chain and side chains of amino acid residues are vulnerable to •OH (*Fig. 4*). Hydroxyl radical initiation of free radical processes causes irreversible damage to protein, including cross-linking, polypeptide chain disruption, and oxidative deamination of lysine and arginine, along with proline and glutamic acid degradation [60, 61]. The aforementioned modifications result in a higher relative carbonyl content within the proteins. Such carbonylation is referred to as direct or primary carbonylation, because the carbonyl groups are formed as a result of oxidation of the polypeptide itself. The involvement of O_2_ •- in this process stems from the typical Haber-Weiss reaction-mediated genesis of •OH (O_2_ •- + H_2_O_2_ → O_2_ + •OH + OH-), a reaction catalyzed by iron and copper ions (Fenton reaction).

The inherent instability of singlet oxygen results in its immediate interaction with carbon-carbon double bonds within lipids, proteins, and carotenoids. In proteins, tryptophan residues constitute its principal target.

Given the substantial reactivity and lack of selectivity exhibited by O_2_ •-, •OH, and 1O_2_ with biomolecules, the primary defense mechanism involves preventing the formation of and eliminating these ROS. Thus, superoxide dismutases, present in all cell compartments, catalyze the conversion of O_2_ •- to H_2_O_2_ whereas carotenoids physically quench 1O_2_.

**Fig. 5:**
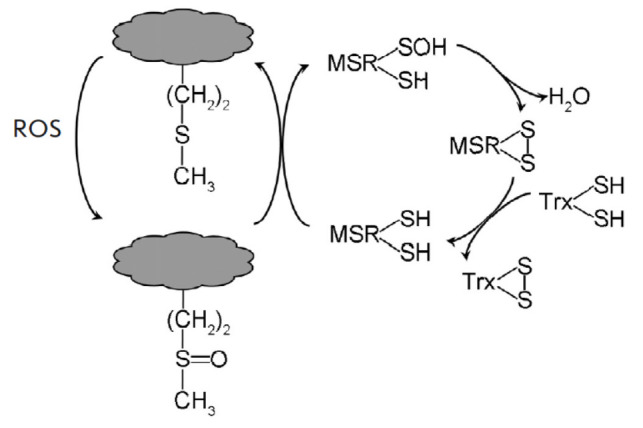
Reduction of oxidized methionine with methionine sulfoxide reductase (MSR)

Hydrogen peroxide has proven to be a useful reagent for the highly selective and reversible redox modification of proteins [65, 66, 67]. Notably, it selectively oxidizes methionine and cysteine residues within living cells [13]. The single-step oxidation of methionine (*Fig. 5*) yields methionine sulfoxide, thereby inhibiting the proteins biological activity. The reduction of methionine sulfoxide is catalyzed by methionine sulfoxide reductases (MSRs). Plant MSRs are characterized by a catalytic site containing two cysteine residues [68]. One cysteine (catalytic) is in the form of the thiolate anion (S-) and is converted into sulfenic acid (SOH), reducing methionine sulfoxide. The other (resolving cysteine) interacts with SOH, which leads to the formation of a disulfide bond. The regeneration of enzymes utilizes Trx, while the Trx regeneration is facilitated by NADPH-dependent or ferredoxin-dependent thioredoxin reductases, as described below. In plant cells, MSRs are located in the cytoplasm, mitochondria, plastids, and endoplasmic reticulum [68]. The methionine sulfoxide/MSR system is often regarded as an “emergency discharge” that channels the ROS attack in the repairable direction [69].

Protein oxidation mediated by ROS, unlike carbonylation with carbohydrate and lipid metabolism byproducts, frequently exhibits reversibility and regulatory functions. These modifications involve a close interplay between spontaneous and enzymatic processes. These reactions collectively comprise a complex network vital to living cells and comparable in significance to reversible protein phosphorylation. This justifies considering ROS as key signaling molecules in various signaling pathways, including those involved in the stress response [70, 71, 72, 73].

## OXIDATION OF CYSTEINE RESIDUES IN PROTEINS

The thiol group of cysteine SH can undergo a range of significant modifications, including oxidation to sulfenic, sulfinic, and sulfonic acids (SOH, SO_2_H, and SO3H, respectively), disulfide bond formation (intraor intermolecular), glutathionylation [74], and persulfidation (interaction with hydrogen sulfide) [75].

Under stress conditions, any SH group in proteins can be oxidized to sulfenic acid by various ROS, including H_2_O_2_ at elevated concentrations [13]. Under favorable conditions, ROS are primarily targeted at dissociated SH-groups, namely, thiolate S- anions. Under physiological conditions, the SH group of cysteine is not dissociated: it has a pKa equal to 8.3. However, a number of proteins contain SH groups that have a pKa below 7 in their microenvironment and dissociate at physiological pH values. These are primarily PRX, glutathione peroxidases (GPX), glutaredoxins (Grx), Trx, and MSR.

**Fig. 6:**
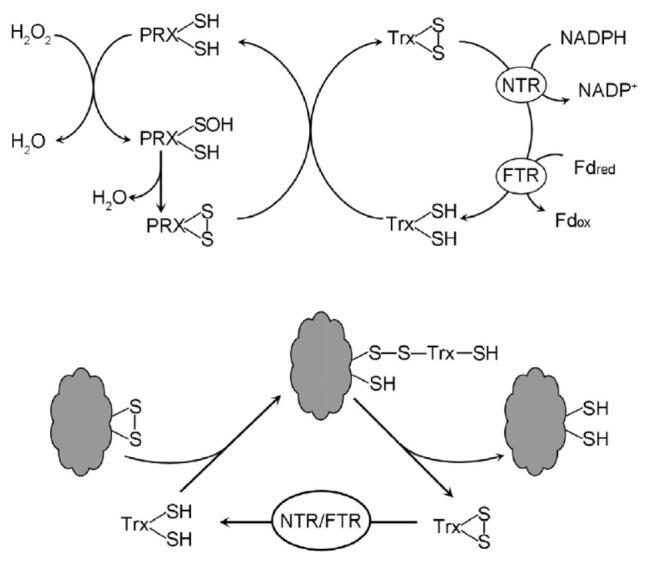
Catalytic cycles of peroxiredoxins (PRX) and thioredoxins (Trx). NTR and FTR are NADPH–dependent and ferredoxin-dependent thioredoxin reductases, respectively. Fdred – reduced ferredoxin, Fdox – oxidized ferredoxin

H_2_O_2_ utilization involves thiol peroxidases, PRX and GPX, which thiolate anion is directly oxidized to sulfenic acid. Plants, in contrast to animals, exhibit diminished GPX activity yet display a diverse array of active PRXs [76, 77]. The interaction between the sulfenic acid and the resolving thiol group in a stand- Fig. 6. Catalytic cycles of peroxiredoxins (PRX) and thioredoxins (Trx). NTR and FTR are NADPH–dependent and ferredoxin-dependent thioredoxin reductases, respectively. Fdred – reduced ferredoxin, Fdox – oxidized ferredoxin ard 2Cys-PRX leads to the formation of an intramolecular disulfide bond (*Fig. 6*).

2Cys-PRX reduction by Trx proceeds via mixed disulfide bond formation. Trx-mediated reduction of disulfide bonds occurs not only in PRX, but also in numerous other proteins residing within diverse cellular compartments, including the cytoplasm, nucleus, plastids, mitochondria, endoplasmic reticulum, and cell wall [78, 79]. The reduction of oxidized Trx is catalyzed by Trx reductases. In plants, these enzymes are represented by NADPH-dependent flavin NTRs and ferredoxin-dependent FTRs with iron-sulfur clusters [4Fe-4S] in their active site, as well as redox-active S-S bonds. Additionally, there is NADPH-dependent NTRC, which assumes the roles of Trx and NTR.

All the reviewed proteins possess redox-sensitive cysteine residues which mediate their involvement in the diverse processes governing the redox metabolism of all living organisms, including plants.

## GLUTATHIONYLATION OF PROTEINS

**Fig. 7:**
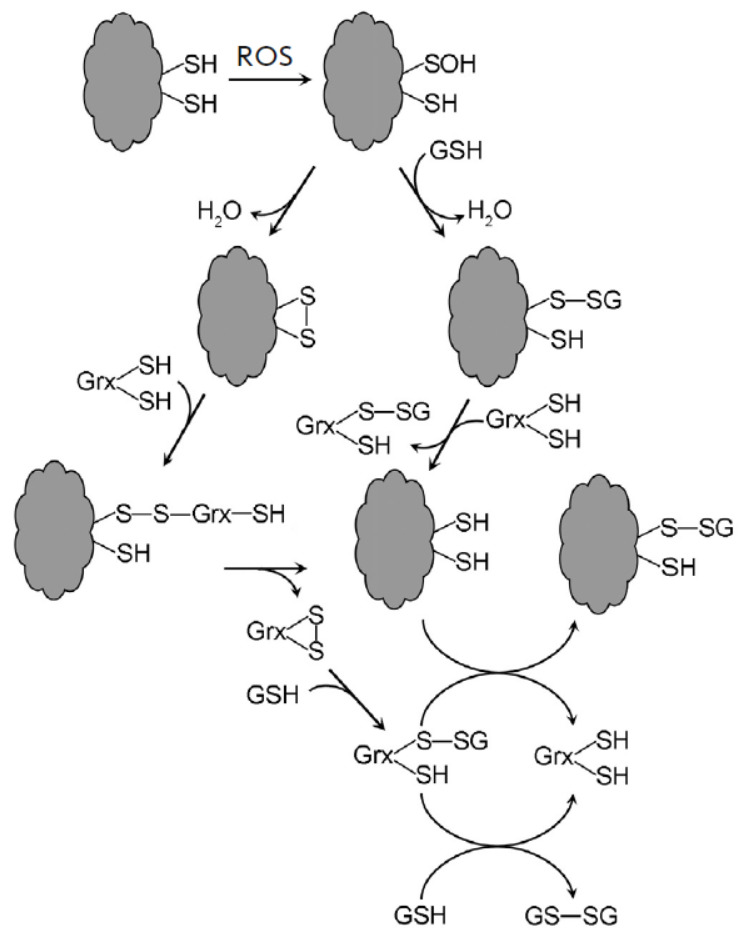
Glutathionylation and deglutathionylation of proteins. Grx – glutaredoxins

Glutathionylation predominantly targets Grx, which catalytic cycle involves such modification of the thiolate anion (*Fig. 7*). Nonetheless, under conditions of oxidative stress, other proteins are also glutathionylated. More than 2,000 glutathionylation sites have been identified within the human proteome [82]. The -S**˙**, -S-, -SOH protein groups exhibit susceptibility to glutathionylation [74]. Glutathionylation is not solely mediated by GSH but also by GSSG, which accumulates under conditions of stress. The glutathionylation of SOH is regarded as a way to prevent the progression of irreversible thiol group oxidation.

Protein deglutathionylation is carried out by Grx, although under stress conditions, they may, in contrast, act as agents of glutathionylation. Glutathionylation, therefore, is a reversible modification that typically inhibits the function of protein. The primary enzymatic targets in plants are cytoplasmic glyceraldehyde-3-phosphate dehydrogenase (GAPDH), other glycolytic enzymes, chloroplast β-amylases, and mitochondrial glycine decarboxylase [74].

## FUNCTIONS OF REDOX MODIFICATIONS OF PROTEIN SULFHYDRYL GROUPS

The principal regulatory mechanism of ROS involves the modification of the target protein thiol groups via S-sulfenylation, S-nitrosylation, and S-glutathionylation. The oxidation of the thiol groups to sulfinic and sulfonic acids typically results in irreversible damage to protein function [83, 84].

**Oxidative protein folding**

Most proteins in the cytoplasm, nucleoplasm, and organelles contain reduced SH groups of cysteine. The process of oxidative folding, which involves the formation of disulfide bridges between the cysteine residues of newly synthesized proteins, is localized in the endoplasmic reticulum, Golgi apparatus, mitochondrial intermembrane space, and thylakoid lumens [85]. The most thoroughly investigated process is oxidative folding in the endoplasmic reticulum lumen. This process affects proteins possessing an N-terminal signal sequence, enabling them to co-translationally enter the endoplasmic reticulum and follow the secretory pathway to the vacuole, cell wall, and plasma membrane [86, 87]. It has been suggested that the stabilization of the native protein conformation in such oxidative compartments is the principal role of disulfide bonds [88].

**Fig. 8:**
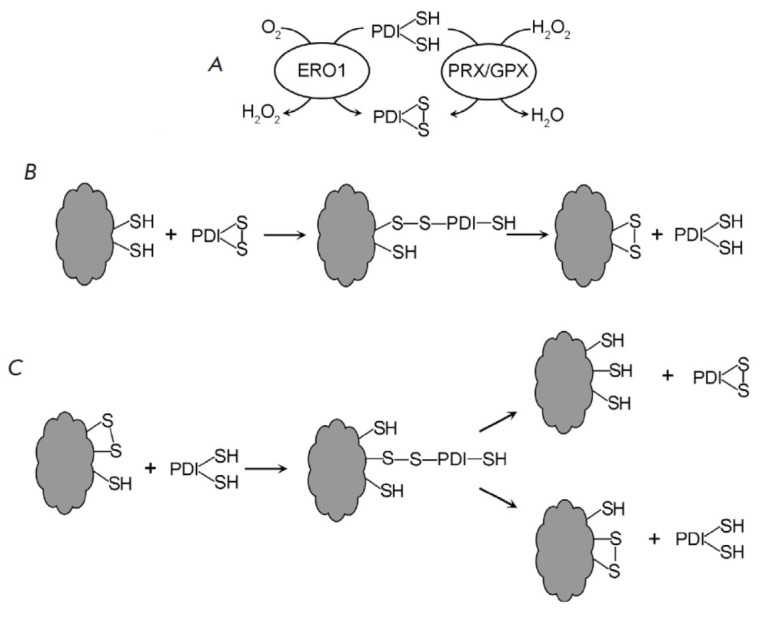
Oxidative folding of proteins in the endoplasmic reticulum lumen. (*A*) Oxidation of protein disulfide isomerase (PDI) under the action of thiol oxidase (ERO1) and thiol peroxidases (PRX and GPX), (*B*) Formation of disulfide bonds by the oxidized form of PDI, (*C*) Isomerization and reduction of disulfide bonds by the reduced form of PDI

Protein disulfide isomerase (PDI) with two cysteine residues per each of its two active sites [89] is the central catalyst for oxidative folding. The presence of a multicomponent redox system within the endoplasmic reticulum lumen results in a dynamic equilibrium, where PDI exists in both the oxidized and reduced forms (*Fig. 8*). PDI oxidation is facilitated by flavin-containing thiol oxidase ERO1 (endoplasmic reticulum oxidoreductin), which utilizes molecular oxygen and produces H_2_O_2_. H_2_O_2_ removal may be achieved through either aquaporin-mediated cytoplasmic diffusion or via thiol peroxidases situated within the endoplasmic reticulum lumen [77]. Furthermore, similar to mammals, plants possess QSOX, a thiol oxidase that combines the functionalities of ERO1 and PDI through O_2_-dependent oxidation of cysteine residues within nascent substrate proteins [90].

Disulfide bond formation requires PDI in its oxidized state (*Fig. 8B*). During oxidative protein folding, PDI is reduced, contributing to the reduced/oxidized PDI balance. A critical component of this balance is the GSH/GSSG redox buffer within the endoplasmic reticulum. The reduction of PDI is possible at the expense of GSH. The reduced PDI facilitates isomerization and reduction of disulfide bonds (*Fig. 8C*).

**Redox regulation of enzyme activity**

Spontaneous and enzyme-controlled oxidative modifications of SH groups affect the conformation of proteins and thereby change their catalytic activity, localization, and ability to protein-protein interact. The cytoplasmic GAPDH in mammals appears to be the most thoroughly researched enzyme in this regard [91]. The active center of this enzyme contains an SH-group with an acid dissociation constant pKa = 6, which is in the form of a thiolate anion (-S-) and exhibits the properties of a strong nucleophile. Oxidation, glutathionylation, and S-nitrosylation of the thiolate anion inhibits GAPDH catalytic activity. Nuclear translocation of oxidized GAPDH initiates the apoptotic pathway. Plant cells possess both cytoplasmic NAD-dependent and plastid NADPH-dependent GAPDH enzymes, both highly sensitive to ROS [92].

Much research has explored the oxidative modifications of catalases in mammals. While oxidation inhibits the catalytic activity of these peroxisomal enzymes, it allows them to participate in protein-protein interactions, enter the nucleus, and influence gene expression. Plant studies have shown similar results [93].

Oxidative stress significantly impacts aconitase, a Krebs cycle enzyme, by oxidizing its iron-sulfur clusters ([4Fe-4S]2+ → [3Fe-4S]+) and sulfhydryl groups (SH → SOH) [94]. Glucose-6-phosphate dehydrogenase, an enzyme in the pentose phosphate pathway, is especially vulnerable to ROS [95].

Redox balance is critical for the processes within chloroplasts. Redox regulation plays a significant role in chlorophyll biosynthesis [96, 97]. This process is known to be controlled by NTRC, a C-type NADPHdependent Trx reductase that combines the functions of Trx and Trx reductase, since unlike classical Trx reductases, the activity of this enzyme affects a wide range of proteins, not just Trx. NTRC maintains the reduced state of the SH-groups of the CHLI subunit of Mg-chelatase, one of the key enzymes of chlorophyll biosynthesis, as well as that of ADP-glucose pyrophosphorylase, an enzyme that determines the rate of starch biosynthesis. Inhibiting NTRC thus impairs chlorophyll and starch biosynthesis [98].

The presence of Trx and its reductases in chloroplasts is necessary in order to activate ribulose bisphosphate carboxylase and other enzymes in the Calvin cycle in response to light [98].

The examples above are all cases where the oxidation of SH groups inhibits enzyme activity. There is less information about the activation of enzymes by the oxidation of SH groups. For example, Arabidopsis ascorbate peroxidase is activated if the SH-group of Cys82 is glutathionylated or is involved in S-S-binding [99]. Dimerization of γ-glutamyl-cysteine synthetase due to the formation of S-S bonds leads to the activation of this key enzyme of GSH biosynthesis [74].

**Signaling role of oxidative modifications of protein thiol groups**

A significant number of components within plant signaling pathways are easily modified by oxidation. For example, the ABA receptor PYR1 and the negative regulators of ABA signaling (ABI1 and ABI2) are inactivated upon oxidation of thiol groups [100]. Salicylic acid (SA) signaling is significantly influenced by redox regulation [101]. The signaling regulator NPR1 (a coactivator of SA-dependent gene transcription) is known to reside in the cytoplasm in an oligomeric form supported by S-S-bridges in the absence of SA. This oligomeric state is reinforced by S-nitrosylation [102]. Pathogen attack triggers SA synthesis, causing oxidative stress, which the plant compensates for by boosting antioxidant defenses, including Trx activation [103]. Thioredoxin-mediated reduction of disulfide bonds in NPR1 leads to oligomer dissociation and nuclear translocation of dimers. These dimers then interact with TGA transcription factors to activate the transcription of pathogenesis-related (PR) genes [104, 105].

The activation of the MAP kinase cascade by ROS is a well-understood phenomenon in animal models. Central to this process is the ASK1, a MAP3K which remains inactive upon binding to reduced Trx. Oxidative stress induces Trx oxidation, disrupting its ASK1 interaction, which subsequently promotes ASK1 dimerization, autophosphorylation, and activation [106]. This is how the MAP kinase cascade is triggered. Plant serine-threonine protein kinase OXI1 (oxidative stress-inducible) becomes activated in response to the oxidative stress induced by a pathogen attack or heavy metal poisoning, subsequently triggering MAPK3/6 activation [107]. However, it is not clear at what level this kinase activates the MAP-kinase cascade: whether it does so via activating MAP3K, MAP2K, or MAPK directly.

Over two decades ago, the first empirical data confirming the existence of ROS-activated cation channels in plants were reported [108, 109]. Currently, Demidchik et al. [110, 111] are developing the concept of the so-called ROS-Ca^2+^-hub, a signaling center in the plasma membrane of the plant cell mediating not only stress reactions, but also the switching on the complex programs of plant development. The activation of Ca^2+^-permeable cation channels, triggered by elevated •OH production in the cell wall, facilitates the cellular uptake of Ca^2+^ and the release of K+. Elevated cytosolic Ca^2+^ concentrations initiate signaling and regulatory cascades within the plant cell [111]. Furthermore, the activation of these channels may be modulated by phosphorylation catalyzed by the protein kinase HPCA (hydrogen peroxide calcium). Within the family of receptor kinases, HPCA is distinguished by its extracellular domain, which contains several redox-sensitive sulfhydryl groups [112]. Upon their oxidation by apoplastic ROS, the cytoplasmic domain of HPCA undergoes autophosphorylation, resulting in the activation of the enzyme, phosphorylation, and the opening of calcium channels in the plasma membrane [113].

It is known that organelles can send signals about their state of oxidative stress to the nucleus and affect the transcription of nuclear genes. In peroxisomes, this retrograde signaling is associated with catalase dysfunction [114]; in mitochondria, with dysfunction of alternative oxidase [115]. The phenomenon of chloroplast retrograde signaling under oxidative stress has been extensively investigated [101, 107, 116], with important observations in Arabidopsis chlorophyll biosynthesis mutants. These mutants accumulate intermediates possessing photosensitizing properties, resulting in singlet oxygen generation. The chloroplast-derived oxidative stress signal generated by light exposure is communicated to the nucleus through the intermediary action of EXE1 and EXE2 proteins, resulting in the activation of a cell death pathway [117]. Oxidation of Trp643 in Arabidopsis EXE1 by singlet oxygen results in EXE1 hydrolysis via the chloroplast metalloprotease FtsH. Retrograde signaling from chloroplasts to the nucleus, involving singlet oxygen and hydrogen peroxide, is modulated by the GUN1 protein [118]. The chloroplast accumulation of 3-phosphoadenosine-5-phosphate (PAP) has also been shown to mediate redox signaling. PAP accumulates under oxidative stress conditions due to the oxidation and inactivation of PAP kinase SAL, which catalyzes its conversion into AMP [116, 119].

The data presented show that the participation of redox modifications of proteins in plant signaling is often mediated by proteins reversible activation/inactivation, changes in their subcellular localization, and susceptibility to degradation in proteasomes.

## CONCLUSION

The late 20th century witnessed the emergence of proteomics, a field of study focused on the exhaustive characterization of the life cycle of proteins within living organisms. This includes, but is not limited to, post-translational modifications, cellular transport, interactions with other molecules, and the processes of both partial and complete degradation. Posttranslational modifications (PTMs), encompassing phosphorylation, glycosylation, methylation, acetylation, carbonylation, and other types of transformations, are typically analyzed in denatured proteins using a combination of chromatographic fractionation and mass spectrometric identification techniques. Advanced methodologies make it easier to both identify PTMs and better picture their dynamics, influence on the protein localization, degradation rates, and interactions with other biomolecules [120]. This progress has also affected the redox proteomics, particularly the proteomics of thiol groups [121]. This review details the chemistry of extensively studied plant protein redox modifications, offering insights into their potential biological functions. Elucidating the functional role of protein redox modifications represents a critical priority in plant proteomics. Recently, a new informational resource, the Plant PTM Viewer (https://www.psb.ugent.be/PlantPTMViewer), has been developed. The Plant PTM Resource database currently holds information on over 300,000 PTMs across more than 130,000 proteins, encompassing those mentioned in this article.

## Abbreviations

ABA: abscisic acid
AGEs: advanced glycation end products
ALEs: advanced lipoxidation end products
GAPDH: glyceraldehyde-3-phosphate dehydrogenase
GPX: glutathione peroxidase
Grx: glutaredoxin
GSH: glutathione
GSNO: nitrosoglutathione
GSSG: glutathione disulfide
GSNOR: nitrosoglutathione reductase
HNE: 4-hydroxy-2-nonenal
MG: methylglyoxal
MDA: malondialdehyde
MSR: methionine sulfoxide reductase
PDI: protein disulfide isomerase
PRX: peroxiredoxins
PTMs: post-translational modifications of proteins
ROS: reactive oxygen species
SA: salicylic acid
Trx: thioredoxin
